# Delayed Bleeding After Endoscopic Ultrasound‐guided Hepaticogastrostomy due to Pseudoaneurysm Rupture in a Patient Who Underwent Plastic Stent Placement: A Case Report

**DOI:** 10.1002/deo2.70218

**Published:** 2025-10-07

**Authors:** Kohei Takano, Takuya Ishikawa, Kentaro Yamao, Yasuyuki Mizutani, Tadashi Iida, Kota Uetsuki, Yoshihisa Takada, Hiroki Kawashima

**Affiliations:** ^1^ Department of Gastroenterology and Hepatology Nagoya University Graduate School of Medicine Aichi Japan; ^2^ Department of Endoscopy Nagoya University Hospital Aichi Japan

**Keywords:** endoscopic ultrasound‐guided hepaticogastrostomy, hemobilia, plastic stent, pseudoaneurysm, transcatheter arterial embolization

## Abstract

Endoscopic ultrasound‐guided hepaticogastrostomy (EUS‐HGS) is a useful alternative treatment for endoscopic retrograde cholangiopancreatography (ERCP) failure. However, serious complications sometimes occur. Bleeding is an early complication that occurs during puncture; however, there have been some reports of late‐onset rupture of a pseudoaneurysm. These reports describe cases of patients who underwent metal stent placement. Herein, we report the first case of pseudoaneurysm formation after plastic stent placement via EUS‐HGS. The patient was a 75‐year‐old man with obstructive jaundice due to pancreatic head cancer. ERCP was unsuccessful, and EUS‐HGS was performed with plastic stent placement from B3. The patient subsequently experienced repeated HGS stent failure within a short period, and the plastic stent was replaced each time. No metal stents were placed during treatment. 106 days after EUS‐HGS, the patient presented with hematochezia and shock, and contrast‐enhanced computed tomography suggested the rupture of a pseudoaneurysm in the left hepatic artery branch. Emergency angiography revealed that the pseudoaneurysm originated from the A2+3 branch of the left hepatic artery, and embolization was performed. Subsequently, there has been no recurrence of bleeding, and the patient was eligible for chemotherapy to treat pancreatic cancer.

## 1 Introduction

During the management of pancreatic cancer, when jaundice occurs due to bile duct obstruction, endoscopic drainage should be considered [1]. Transpapillary biliary drainage is often the first choice, but this approach could be difficult due to invasion of the papilla or duodenum. Endoscopic ultrasound (EUS)‐guided drainage/anastomosis is a useful treatment in such cases, but complications occur in 15%–24% of patients [2]. Bleeding is an early complication that often occurs due to a puncture. However, herein, we report a case in which a pseudoaneurysm formed due to plastic stent placement by EUS‐HGS, leading to delayed bleeding induced by pseudoaneurysm rupture.

## 2 Case Report

**FIGURE 1:**
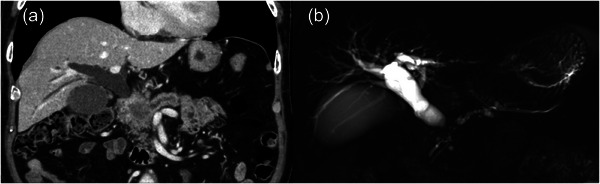
Contrast‐enhanced computed tomography (CE‐CT) and magnetic resonance cholangiopancreatography (MRCP) images at diagnosis. (a) CE‐CT image showing a pancreatic head tumor and dilation of the main pancreatic duct on the caudal side. The distal bile duct is obstructed by the pancreatic head tumor. It was considered borderline resectable according to the NCCN clinical practice guideline version 2.2021. (b) MRCP image showing dilatation of the main pancreatic duct distal to the pancreatic head tumor. The intrahepatic bile ducts were dilated up to 7 mm, and the extrahepatic bile duct was dilated up to 14 mm.

The patient was a 75‐year‐old man. He presented with jaundice 2 weeks before admission. Abdominal computed tomography (CT) revealed a pancreatic head tumor and dilation of the bile duct upstream of the lesion (Figure 1a). Magnetic resonance cholangiopancreatography revealed dilatation of the main pancreatic duct and the bile duct distal to the mass lesion (Figure 1b).

**FIGURE 2:**
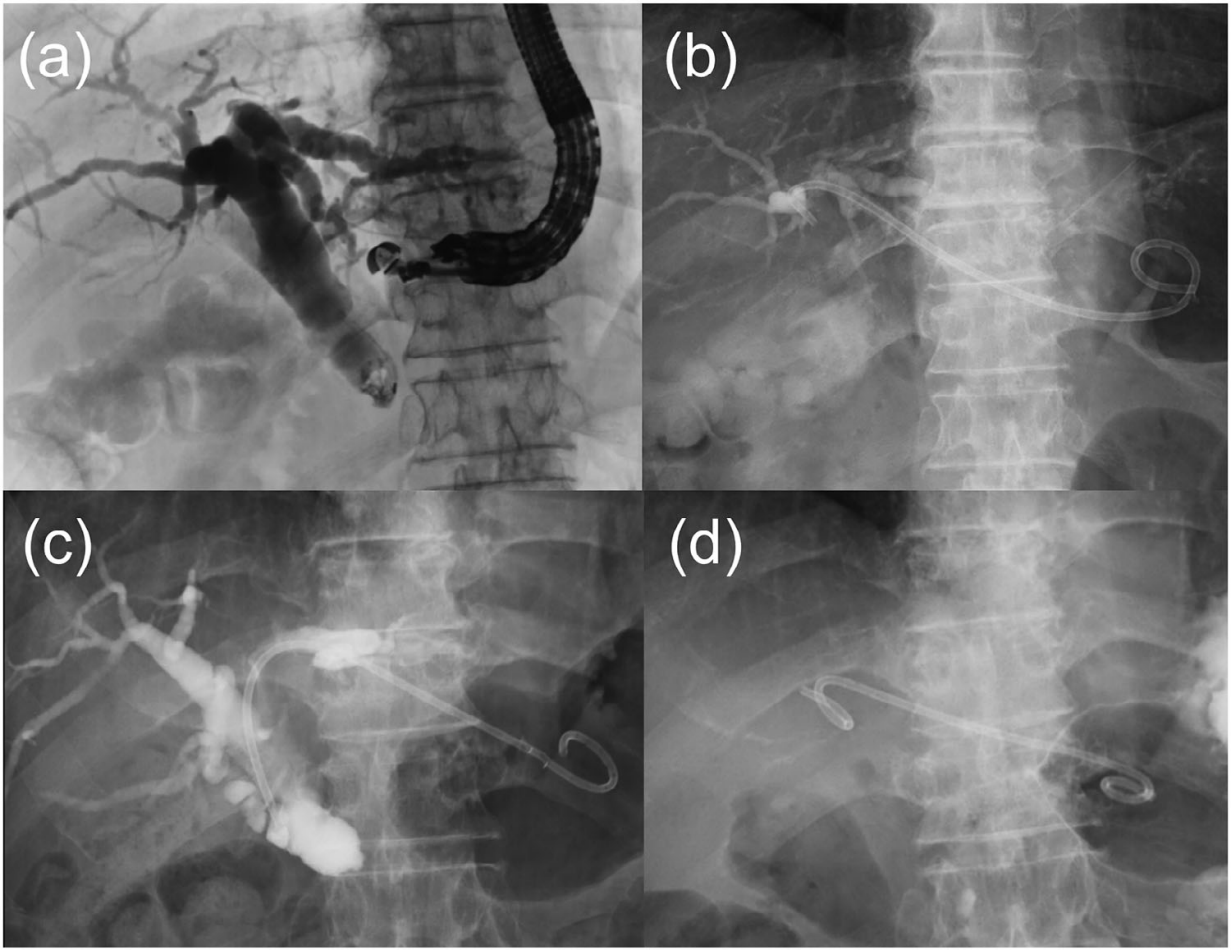
Endoscopic ultrasound‐guided hepaticogastrostomy (EUS‐HGS). (a) Fluoroscopy during EUS‐HGS. There was an obstruction in the distal bile duct, and dilatation of the bile duct upstream was observed. The area distal to the bile duct obstruction was not contrasted. (b) The plastic stent was placed via the stomach from B3. The end of the stent was located at the left hepatic duct. (c) On day 19 after EUS‐HGS, the HGS stent was exchanged. A 7‐Fr single‐pigtail plastic stent was placed with the tip positioned in the common bile duct. (d) On day 77 after EUS‐HGS, a 7‐Fr double‐pigtail plastic stent was placed for the HGS stent.

A diagnosis of adenocarcinoma was obtained via EUS‐guided tissue acquisition. To alleviate jaundice, ERCP was attempted, but it was unsuccessful because bile duct catheterization was difficult. EUS‐HGS was performed as an alternative treatment. GF‐UCT240 (Olympus Corporation, Tokyo) was used, and transgastric scanning revealed dilated B3 bile ducts (7 mm, Figure S1). A 19‐gauge FNA needle (EZ shot 3 Plus; Olympus Corporation, Tokyo) was used to puncture the bile duct. The puncture of the bile duct was performed only on the anterior wall, and the posterior wall was not punctured. The puncture route was dilated using a 4‐mm tapered tip balloon (REN Type IT; Kaneka Medix Co., Ltd., Osaka), and a 7‐Fr single‐pigtail plastic stent (Through & Pass TYPE IT; Gadelius Medical Co., Ltd., Tokyo) was placed. The tip of the stent was positioned on the left hepatic duct (Figure 2a,b). After EUS‐HGS, the patient's jaundice improved. On day 9 after EUS‐HGS, diagnostic laparoscopy showed positive peritoneal lavage cytology, indicating inoperability. On day 19, the patient developed a fever, likely due to HGS stent failure. Because the stent tip remained in the left hepatic duct and the stent became bent in the stomach, poor stability was anticipated. Therefore, the same stent was placed with the tip positioned in the common bile duct (Figure 2c). The cholangitis improved, and chemotherapy (gemcitabine plus nab‐paclitaxel) was initiated.

**FIGURE 3:**
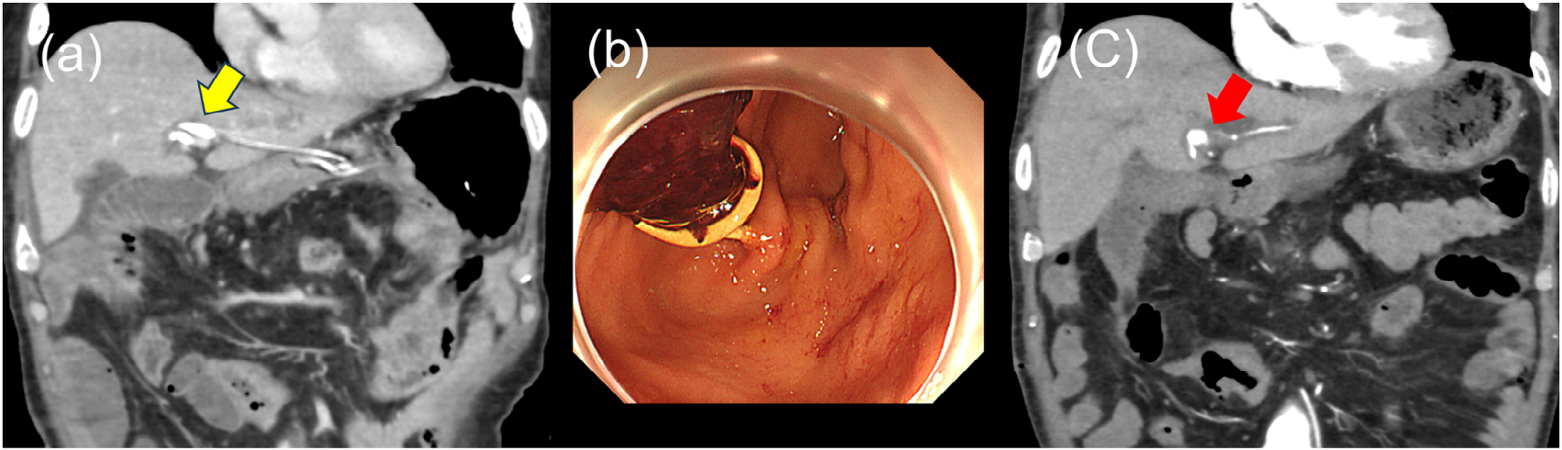
Images of hemobilia. (a) Computed tomography (CT) image of hemobilia at day 103. The bile duct shows patchy high density due to hemobilia. The tip of the hepaticogastrostomy (HGS) stent was coiled in a pigtail shape near B2+3 (yellow arrow). (b) Endoscopic findings. Blood clots were attached to the HGS stent, and blood flow from the stent's side hole was observed. (c) CT when the patient was in shock at day 106. The bile duct stent was removed. A pseudoaneurysm formed at the location of the HGS stent tip (red arrow).

**FIGURE 4:**
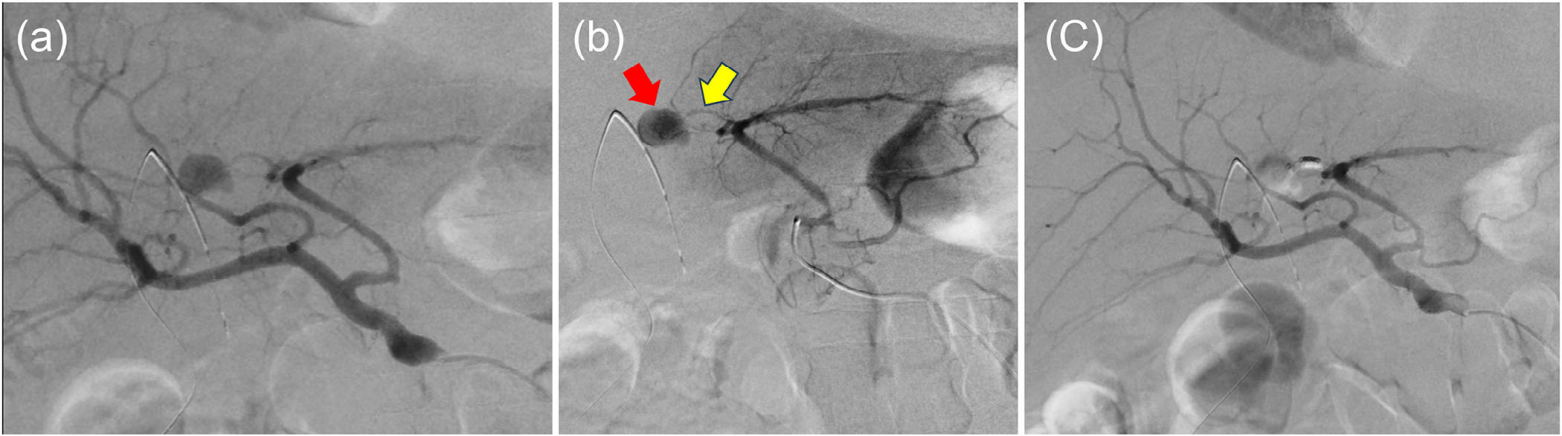
Transcatheter arterial embolization (TAE) (a) Digital subtraction angiography (DSA) of the common hepatic artery. Extravasation originating from the left hepatic artery or the middle hepatic artery was suspected. (b) DSA image of the left hepatic artery. Extravasation (red arrow) originating from the A2+3 subbranch (yellow arrow) is observed. (c) DSA image of the common hepatic artery after coil embolization. Extravasation has disappeared.

On day 77, jaundice recurred. The plain CT scan revealed a high‐density area in the bile duct, suggesting hemobilia. The HGS stent was replaced endoscopically again. At this time, blood flowed into the stomach from the bile duct‐gastric anastomosis. However, since no pseudoaneurysm or extravasation was observed on CT, the cause of hemobilia was considered the fragility of the bile duct wall due to chronic inflammation and HGS stent. So we decided to perform stent replacement only. To prevent dislocation, a 7‐Fr double‐pigtail plastic stent (Through & Pass Double Pigtail; Gadelius Medical Co., Ltd., Tokyo) was placed (Figure 2d). The symptoms of cholangitis improved temporarily but subsequently worsened, and endoscopic examination was repeated on day 89. The plain CT scan revealed a high‐density area in the bile duct. No pseudoaneurysm was detected by the contrast‐enhanced CT. At that time, moderate bleeding flowed from the bile duct‐stomach anastomosis into the stomach, and hemobilia was suspected again. Blood transfusion and conservative treatment were attempted, but ongoing hematemesis was observed, and anemia did not improve. Vomiting also occurred due to duodenal stenosis. On day 95, CT images showed the same findings as those on day 89, and hemobilia was suspected. To control cholangitis due to hemobilia and duodenal stenosis, bile duct‐jejunal anastomosis, gastric‐jejunal anastomosis, and cholecystectomy were performed. Thereafter, the patient's condition improved without bleeding symptoms. On day 103 after EUS‐HGS, the patient experienced hematemesis and shock, and an endoscopic examination was performed. Blood clots were found in the stomach, and blood flowed from the bile duct‐gastric anastomosis (Figure 3a,b and Figure S2a,b). The HGS stent was considered the cause of bile duct hemorrhage, and upon removal, no active bleeding was found at the anastomosis site, and the patient was placed under observation. Although there were no signs of rebleeding thereafter, on day 106, the patient again experienced hematochezia and shock. Multiphasic contrast‐enhanced CT revealed a pseudoaneurysm at the tip of the previously placed bile duct stent (Figure 3c and Figure S2c), and bleeding from this site was suspected. Emergency transcatheter arterial embolization (TAE) was performed. Digital subtraction angiography (DSA) revealed bleeding from the A2+3 branch, and the branch was embolized with coils (Figure 4). The patient had a favorable course after TAE, and as of 2 months post‐procedure, chemotherapy was continued without any rebleeding.

## 3 Discussion

This case report highlights a rare complication of EUS‐HGS: delayed bleeding due to rupture of a left hepatic artery pseudoaneurysm.

Pseudoaneurysms and hemobilia are relatively rare complications of endoscopic treatment. The incidence of pseudoaneurysm formation following bile duct stent placement during ERCP has been reported to be 1.2% [3], and diagnosis may be challenging in some cases.

Bleeding associated with EUS‐HGS has been reported in 3.7% of cases [4], but most of these cases are due to vascular damage during puncture, and there are limited reports of delayed bleeding due to pseudoaneurysm rupture.

EUS‐HGS is typically performed by approaching the left hepatic lobe, performing dilation, and placing a stent, which may increase the risk of damage to the left hepatic artery branches.

The mechanism of pseudoaneurysm formation after EUS‐HGS is not fully understood, as few cases have been reported. However, several hypotheses have been proposed, including the formation of a fistula at the antral region, chronic stimulation caused by the movement of the stent due to gastric peristalsis [5], and the possibility that a drill‐type dilator used for tract dilation may contribute to vascular injury [6]. In the present case, EUS‐HGS was performed on the anterior wall of the antrum near the gastric angle, which is susceptible to the effects of gastric peristalsis. It is considered that chronic inflammation weakened the bile duct wall, and that repeated mechanical stimulation by the hepatic end of the stent, particularly the pigtail‐shaped tip, exacerbates this fragility, ultimately resulting in pseudoaneurysm formation. In this case, gastroenterologists and radiologists evaluated the images, but it was difficult to identify the pseudoaneurysm by CT scan on day 103, obtained only in the equilibrium phase. It is possible that the HGS stent obscured the recognition of the pseudoaneurysm. Therefore, it may be considered to temporarily remove the stent and perform imaging evaluations such as multiphasic CT scans, including arterial phase, to identify a pseudoaneurysm, if possible.

Pseudoaneurysm formation or rupture following EUS‐HGS has previously been reported in three cases, all of which involved metal stent placement [5, 6, 7]. To our knowledge, this is the first reported case of pseudoaneurysm formation associated with a plastic stent placed via EUS‐HGS. In addition, a few cases of bile duct hemorrhage following ERCP after plastic stent placement have also been reported [8, 9]. In the case of metal stents, pseudoaneurysms can develop both at the tip and the expansion site due to mechanical stimulation. In contrast, plastic stents cause milder stimulation of the bile duct wall and surrounding arteries at the placement site, but caution is still needed regarding the position of the stent tip. We believe that the pseudoaneurysm most likely formed on day 77 after stent placement, as shown in Figure 3.

TAE, which is minimally invasive and has a high success rate (80%–100%), can also serve as a diagnostic tool and is considered the first‐line treatment for pseudoaneurysm rupture [10].

In conclusion, we report a rare case of delayed bleeding due to rupture of a pseudoaneurysm that developed after plastic stent placement by EUS‐HGS. In cases of recurrent cholangitis or rapid progression of anemia after EUS‐HGS, it is necessary to consider performing a multiphasic CT scan and including pseudoaneurysm formation and rupture in the differential diagnosis. In such cases, it is important to note that pseudoaneurysms may be difficult to identify because of the stent.

## Author Contributions

**Kohei Takano**, **Takuya Ishikawa**, and **Hiroki Kawashima** helped conceptualize the report. **Kohei Takano** helped draft the manuscript. All the authors reviewed and revised the manuscript draft and approved the final version for submission.

## Conflicts of Interest

The authors declare no conflicts of interest.

## Ethics Statement

Approval of the research by an Institutional Review Board: The report was approved by the Ethics Committee at Nagoya University Hospital.

## Consent

Written informed consent was obtained from all patients involved in the study.

## Clinical Trial Registration

N/A.

## Supporting information




**FIGURE S1** EUS‐HGS. (a) EUS image. A dilated B3 bile duct was observed. (b) Color Doppler image. No blood flow signal was observed at the planned puncture line.

**FIGURE S2** Images of hemobilia. (a, b) CT images of hemobilia at day 103. (a) Plain CT. The bile duct shows patchy high density due to hemobilia. (b) Contrast‐enhanced CT revealed no aneurysm. (c) CT when the patient was in shock at day 106. The bile duct stent was removed. A pseudoaneurysm formed at the location of the HGS stent tip (red arrow). The black arrow shows the percutaneous transhepatic biliary drainage tube. This was placed during surgery on day 95 to control cholangitis. The green arrow shows the left hepatic artery. The blue arrow shows the middle hepatic artery.
