# Supplementary figures and images for: Delayed Bleeding After Endoscopic Ultrasound‐guided Hepaticogastrostomy due to Pseudoaneurysm Rupture in a Patient Who Underwent Plastic Stent Placement: A Case Report
Source: DEN Open. 2025 Oct 7;6(1):e70218. doi: 10.1002/deo2.70218 (PMC12504042; doi:10.1002/deo2.70218)

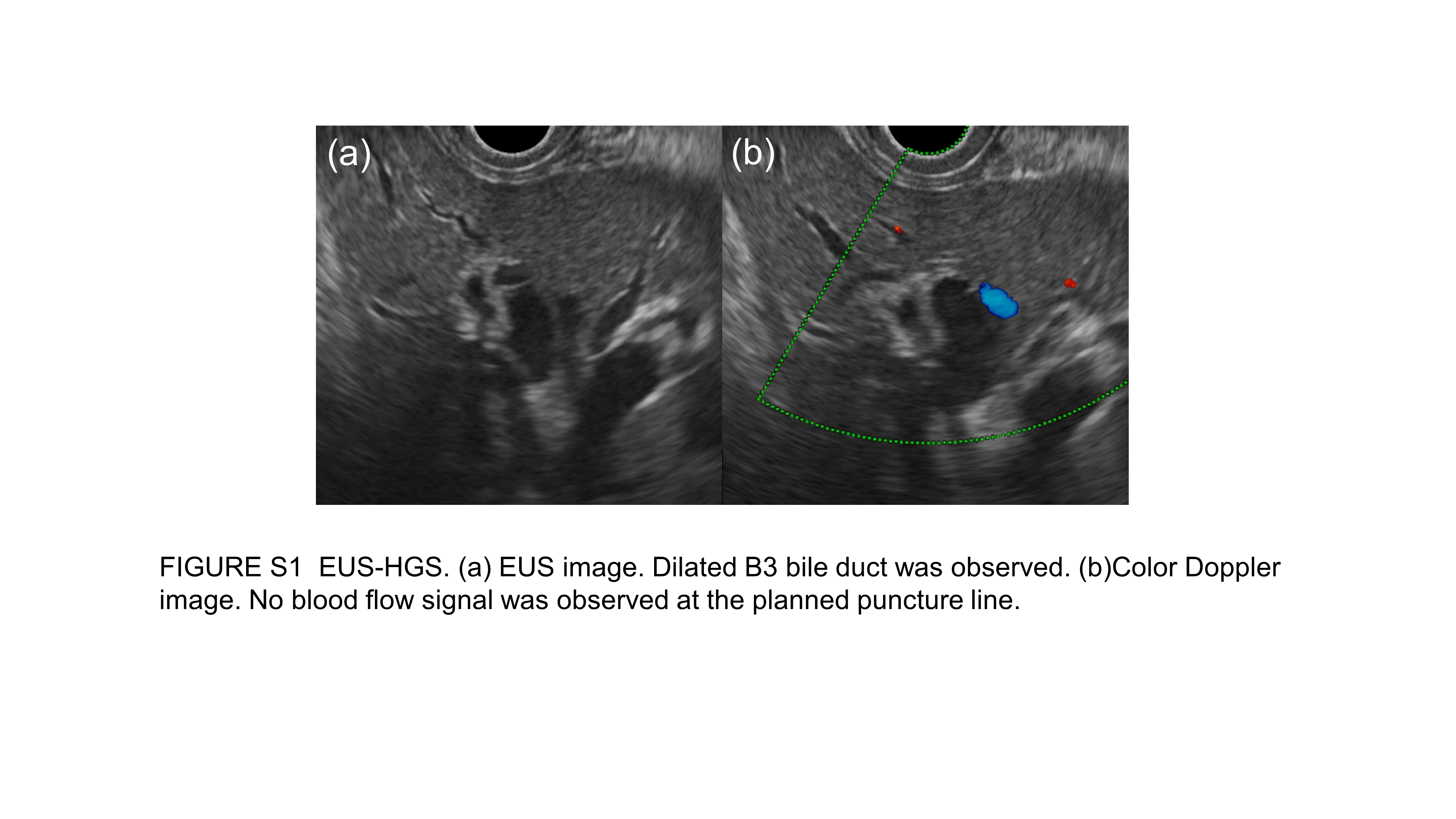

Supplement: Supplementary file 1 — FIGURE S1 EUS‐HGS. (a) EUS image. A dilated B3 bile duct was observed. (b) Color Doppler image. No blood flow signal was observed at the planned puncture line. [file DEO2-6-e70218-s002.tif]

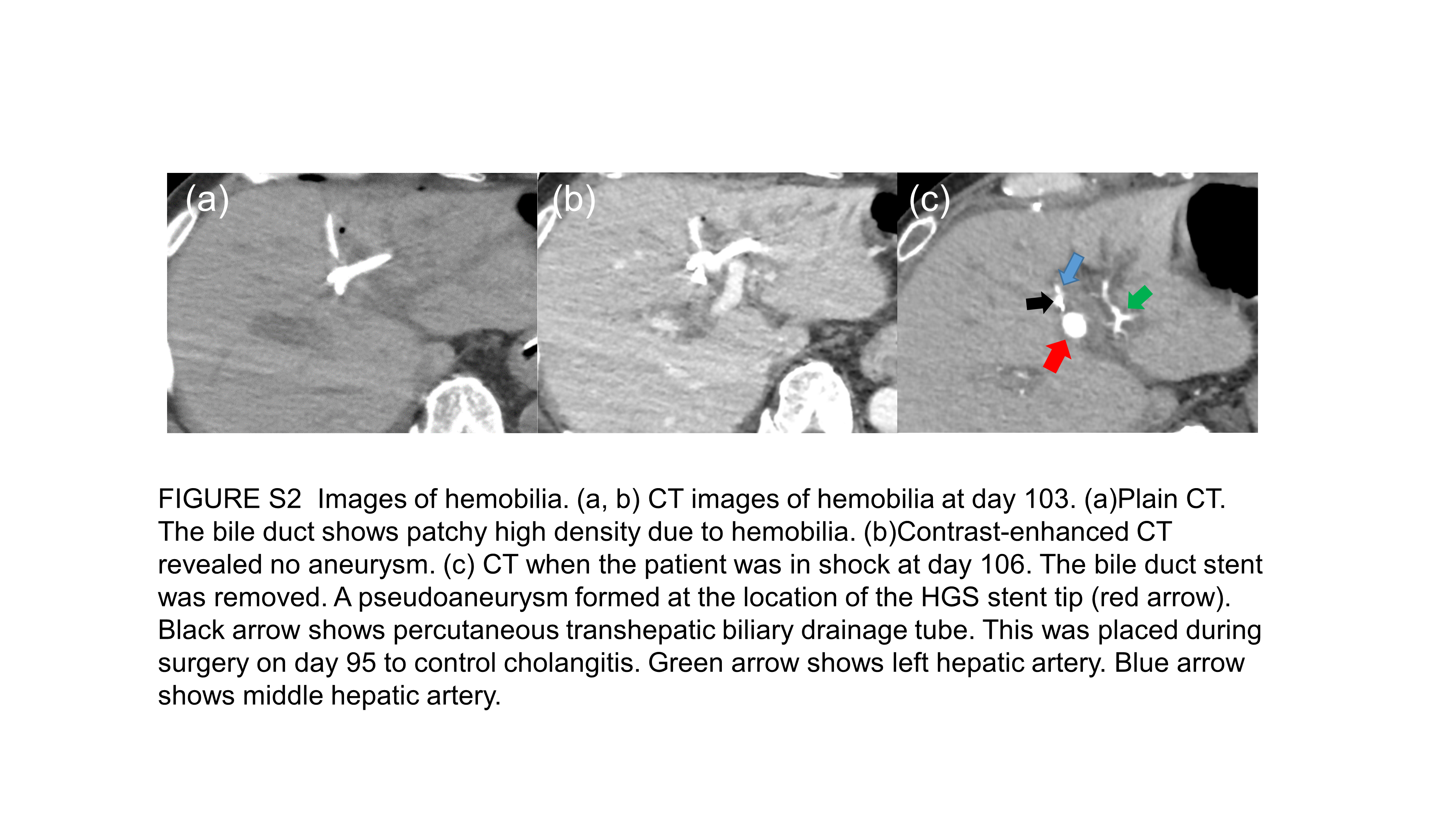

Supplement: Supplementary file 2 — FIGURE S2 Images of hemobilia. (a, b) CT images of hemobilia at day 103. (a) Plain CT. The bile duct shows patchy high density due to hemobilia. (b) Contrast‐enhanced CT revealed no aneurysm. (c) CT when the patient was in shock at day 106. The bile duct stent was removed. A pseudoaneurysm formed at the location of the HGS stent tip (red arrow). The black arrow shows the percutaneous transhepatic biliary drainage tube. This was placed during surgery on day 95 to control cholangitis. The green arrow shows the left hepatic artery. The blue arrow shows the middle hepatic artery. [file DEO2-6-e70218-s001.tif]
